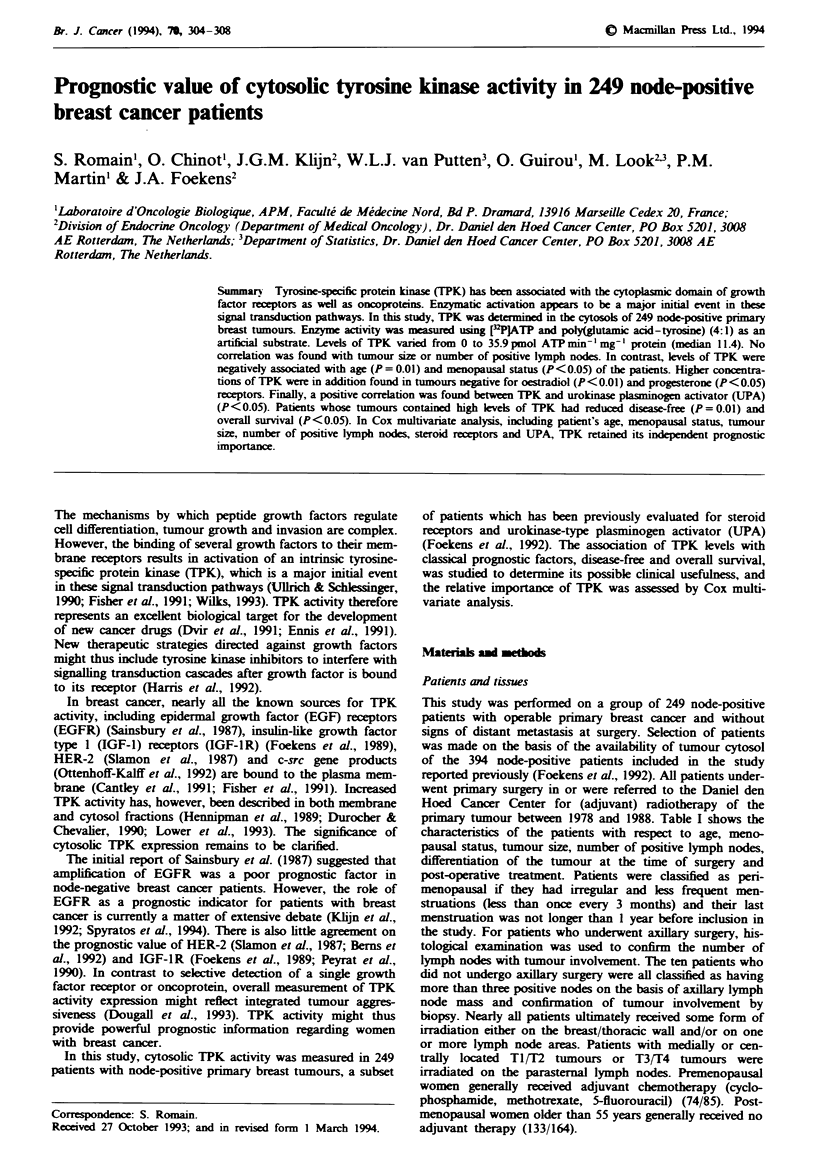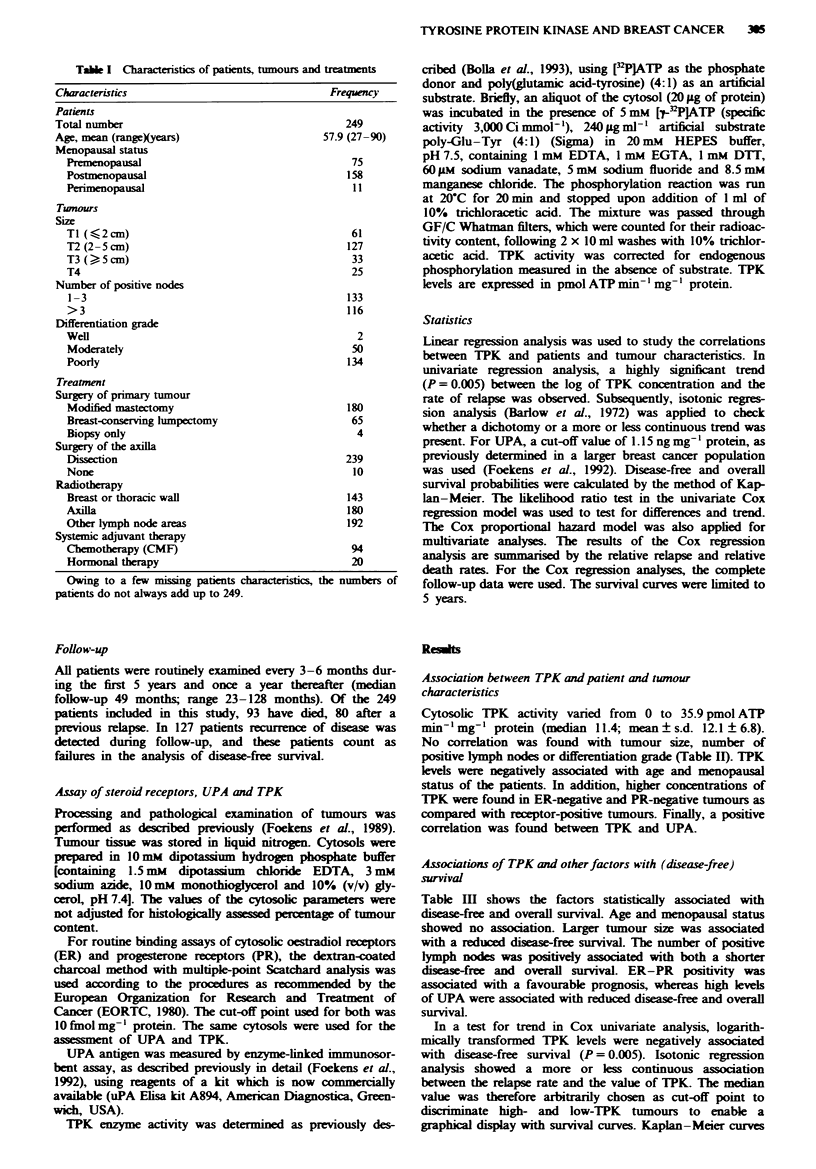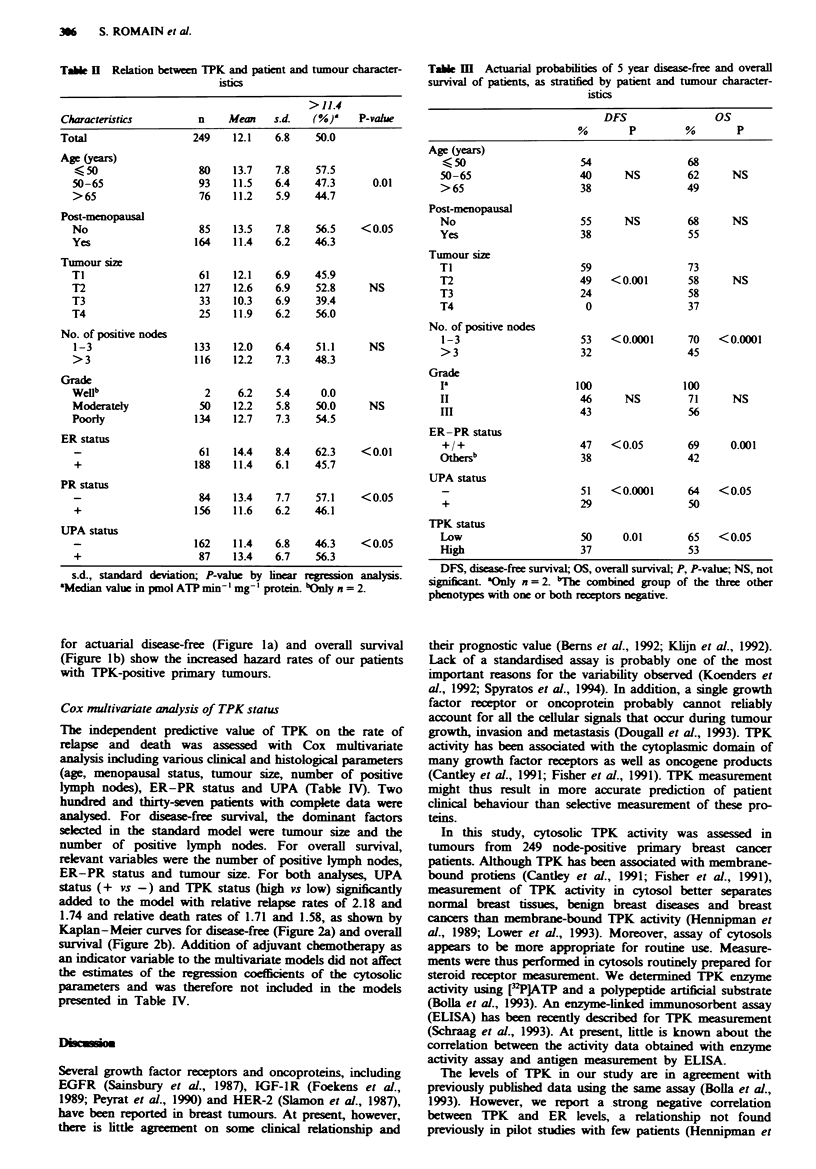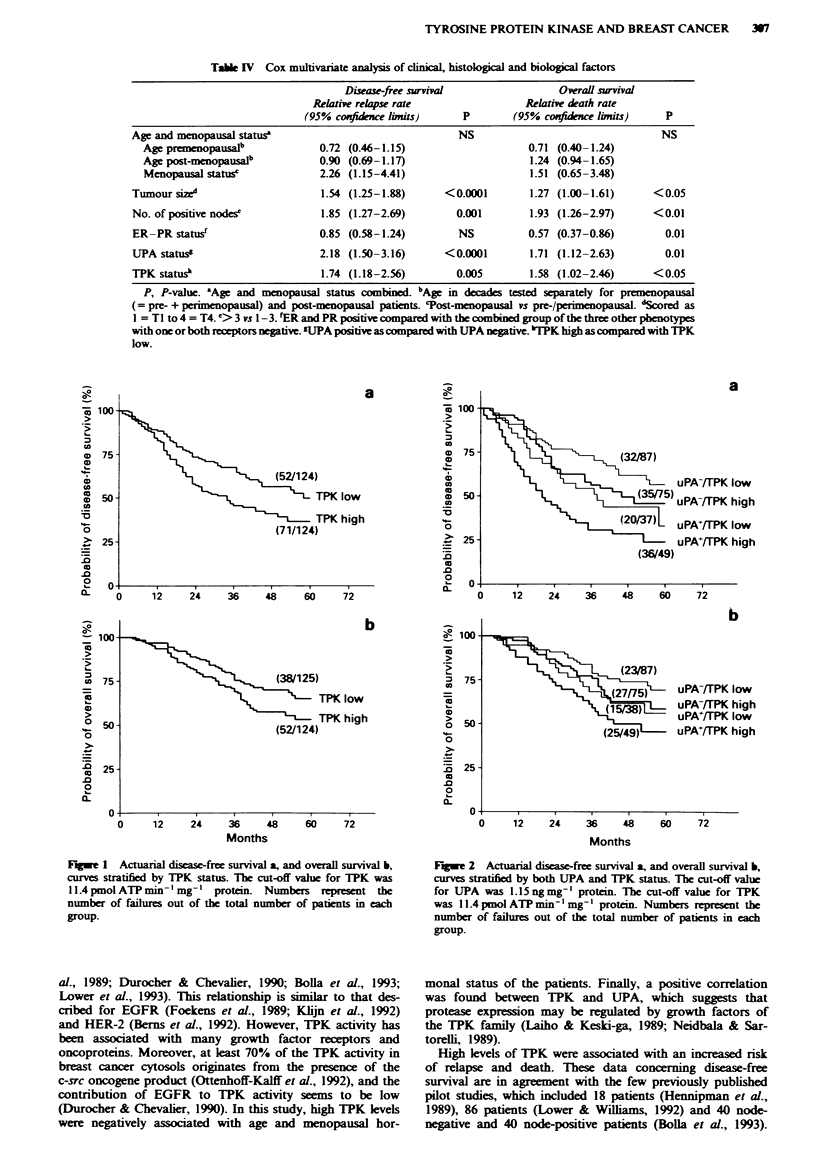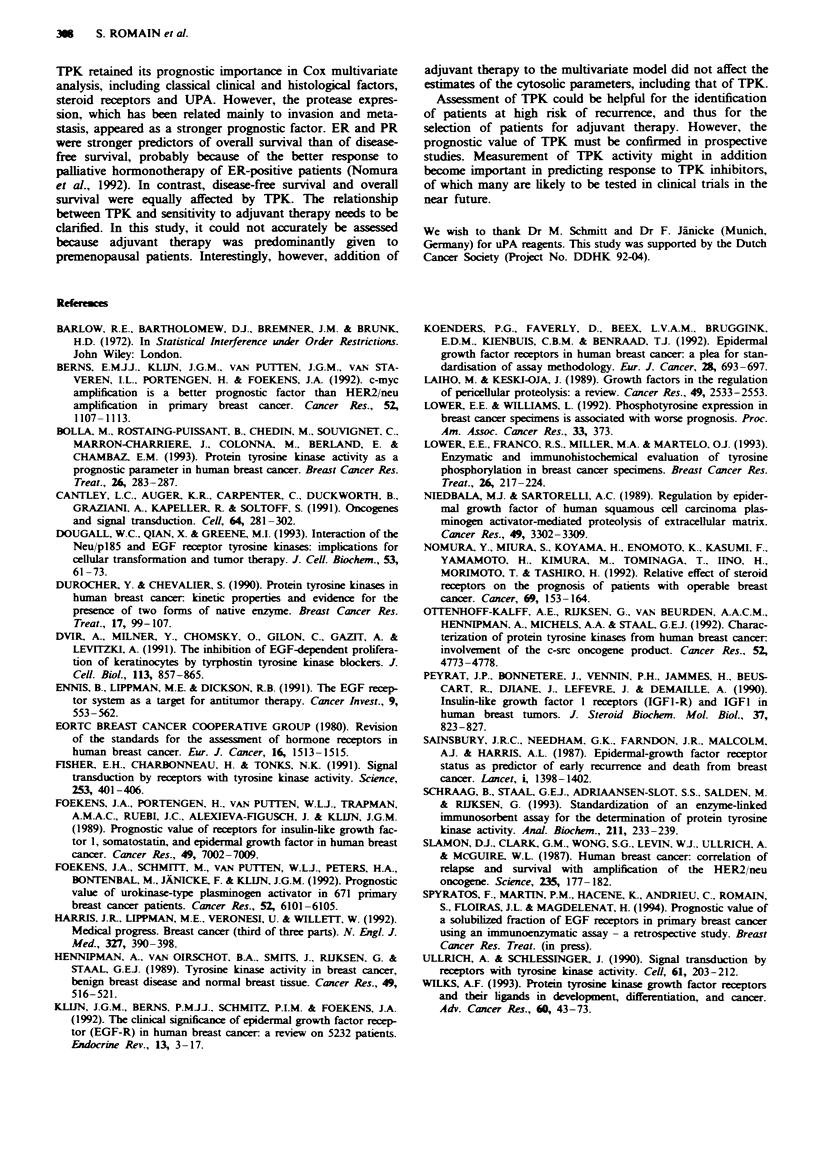# Prognostic value of cytosolic tyrosine kinase activity in 249 node-positive breast cancer patients.

**DOI:** 10.1038/bjc.1994.297

**Published:** 1994-08

**Authors:** S. Romain, O. Chinot, J. G. Klijn, W. L. van Putten, O. Guirou, M. Look, P. M. Martin, J. A. Foekens

**Affiliations:** Laboratoire d'Oncologie Biologique, APM, Faculté de Médecine Nord, Marseille, France.

## Abstract

Tyrosine-specific protein kinase (TPK) has been associated with the cytoplasmic domain of growth factor receptors as well as oncoproteins. Enzymatic activation appears to be a major initial event in these signal transduction pathways. In this study, TPK was determined in the cytosols of 249 node-positive primary breast tumours. Enzyme activity was measured using [32P]ATP and poly(glutamic acid-tyrosine) (4:1) as an artificial substrate. Levels of TPK varied from 0 to 35.9 pmol ATP min-1 mg-1 protein (median 11.4). No correlation was found with tumour size or number of positive lymph nodes. In contrast, levels of TPK were negatively associated with age (P = 0.01) and menopausal status (P < 0.05) of the patients. Higher concentrations of TPK were in addition found in tumours negative for oestradiol (P < 0.01) and progesterone (P < 0.05) receptors. Finally, a positive correlation was found between TPK and urokinase plasminogen activator (UPA) (P < 0.05). Patients whose tumours contained high levels of TPK had reduced disease-free (P = 0.01) and overall survival (P < 0.05). In Cox multivariate analysis, including patient's age, menopausal status, tumour size, number of positive lymph nodes, steroid receptors and UPA, TPK retained its independent prognostic importance.


Br. J. Cancer (1994), 70, 304-308 © Macmillan Press Ltd., 1994

### Prognostic value of cytosolic tyrosine kinase activity in 249 node-positive breast cancer patients

S. Romain[1], O. Chinot[1], J.G.M. Klijn[2], W.L.J. van Putten[3], O. Guirou[1], M. Look[2,3], P.M. Martin[1] & J.A. Foekens[2]

[1]*Laboratoire d'Oncologie Biologique, APM, Faculté de Médecine Nord, Bd P. Dramard, 13916 Marseille Cedex 20, France;* [2]*Division of Endocrine Oncology (Department of Medical Oncology), Dr. Daniel den Hoed Cancer Center, PO Box 5201, 3008 AE Rotterdam, The Netherlands;* [3]*Department of Statistics, Dr. Daniel den Hoed Cancer Center, PO Box 5201, 3008 AE Rotterdam, The Netherlands.*

**Summary** Tyrosine-specific protein kinase (TPK) has been associated with the cytoplasmic domain of growth factor receptors as well as oncoproteins. Enzymatic activation appears to be a major initial event in these signal transduction pathways. In this study, TPK was determined in the cytosols of 249 node-positive primary breast tumours. Enzyme activity was measured using [$^{32}$P]ATP and poly(glutamic acid-tyrosine) (4:1) as an artificial substrate. Levels of TPK varied from 0 to 35.9 pmol ATP $min^{-1} mg^{-1}$ protein (median 11.4). No correlation was found with tumour size or number of positive lymph nodes. In contrast, levels of TPK were negatively associated with age ($P = 0.01$) and menopausal status ($P < 0.05$) of the patients. Higher concentrations of TPK were in addition found in tumours negative for oestradiol ($P < 0.01$) and progesterone ($P < 0.05$) receptors. Finally, a positive correlation was found between TPK and urokinase plasminogen activator (UPA) ($P < 0.05$). Patients whose tumours contained high levels of TPK had reduced disease-free ($P = 0.01$) and overall survival ($P < 0.05$). In Cox multivariate analysis, including patient's age, menopausal status, tumour size, number of positive lymph nodes, steroid receptors and UPA, TPK retained its independent prognostic importance.

The mechanisms by which peptide growth factors regulate cell differentiation, tumour growth and invasion are complex. However, the binding of several growth factors to their membrane receptors results in activation of an intrinsic tyrosine-specific protein kinase (TPK), which is a major initial event in these signal transduction pathways (Ullrich & Schlessinger, 1990; Fisher *et al.*, 1991; Wilks, 1993). TPK activity therefore represents an excellent biological target for the development of new cancer drugs (Dvir *et al.*, 1991; Ennis *et al.*, 1991). New therapeutic strategies directed against growth factors might thus include tyrosine kinase inhibitors to interfere with signalling transduction cascades after growth factor is bound to its receptor (Harris *et al.*, 1992).

In breast cancer, nearly all the known sources for TPK activity, including epidermal growth factor (EGF) receptors (EGFR) (Sainsbury *et al.*, 1987), insulin-like growth factor type 1 (IGF-1) receptors (IGF-1R) (Foekens *et al.*, 1989), HER-2 (Slamon *et al.*, 1987) and c-*src* gene products (Ottenhoff-Kalff *et al.*, 1992) are bound to the plasma membrane (Cantley *et al.*, 1991; Fisher *et al.*, 1991). Increased TPK activity has, however, been described in both membrane and cytosol fractions (Hennipman *et al.*, 1989; Durocher & Chevalier, 1990; Lower *et al.*, 1993). The significance of cytosolic TPK expression remains to be clarified.

The initial report of Sainsbury *et al.* (1987) suggested that amplification of EGFR was a poor prognostic factor in node-negative breast cancer patients. However, the role of EGFR as a prognostic indicator for patients with breast cancer is currently a matter of extensive debate (Klijn *et al.*, 1992; Spyratos *et al.*, 1994). There is also little agreement on the prognostic value of HER-2 (Slamon *et al.*, 1987; Berns *et al.*, 1992) and IGF-1R (Foekens *et al.*, 1989; Peyrat *et al.*, 1990). In contrast to selective detection of a single growth factor receptor or oncoprotein, overall measurement of TPK activity expression might reflect integrated tumour aggressiveness (Dougall *et al.*, 1993). TPK activity might thus provide powerful prognostic information regarding women with breast cancer.

In this study, cytosolic TPK activity was measured in 249 patients with node-positive primary breast tumours, a subset of patients which has been previously evaluated for steroid receptors and urokinase-type plasminogen activator (UPA) (Foekens *et al.*, 1992). The association of TPK levels with classical prognostic factors, disease-free and overall survival, was studied to determine its possible clinical usefulness, and the relative importance of TPK was assessed by Cox multivariate analysis.

Correspondence: S. Romain.
Received 27 October 1993; and in revised form 1 March 1994.

### Materials and methods

### *Patients and tissues*

This study was performed on a group of 249 node-positive patients with operable primary breast cancer and without signs of distant metastasis at surgery. Selection of patients was made on the basis of the availability of tumour cytosol of the 394 node-positive patients included in the study reported previously (Foekens *et al.*, 1992). All patients underwent primary surgery in or were referred to the Daniel den Hoed Cancer Center for (adjuvant) radiotherapy of the primary tumour between 1978 and 1988. Table I shows the characteristics of the patients with respect to age, menopausal status, tumour size, number of positive lymph nodes, differentiation of the tumour at the time of surgery and post-operative treatment. Patients were classified as perimenopausal if they had irregular and less frequent menstruations (less than once every 3 months) and their last menstruation was not longer than 1 year before inclusion in the study. For patients who underwent axillary surgery, histological examination was used to confirm the number of lymph nodes with tumour involvement. The ten patients who did not undergo axillary surgery were all classified as having more than three positive nodes on the basis of axillary lymph node mass and confirmation of tumour involvement by biopsy. Nearly all patients ultimately received some form of irradiation either on the breast/thoracic wall and/or on one or more lymph node areas. Patients with medially or centrally located T1/T2 tumours or T3/T4 tumours were irradiated on the parasternal lymph nodes. Premenopausal women generally received adjuvant chemotherapy (cyclophosphamide, methotrexate, 5-fluorouracil) (74/85). Postmenopausal women older than 55 years generally received no adjuvant therapy (133/164).

**Table I** Characteristics of patients, tumours and treatments

| *Characteristics* | *Frequency* |
|---|---|
| *Patients* | |
| Total number | 249 |
| Age, mean (range)(years) | 57.9 (27-90) |
| Menopausal status | |
| Premenopausal | 75 |
| Postmenopausal | 158 |
| Perimenopausal | 11 |
| *Tumours* | |
| Size | |
| T1 ($\leqslant$2 cm) | 61 |
| T2 (2-5 cm) | 127 |
| T3 ($\geqslant$5 cm) | 33 |
| T4 | 25 |
| Number of positive nodes | |
| 1-3 | 133 |
| >3 | 116 |
| Differentiation grade | |
| Well | 2 |
| Moderately | 50 |
| Poorly | 134 |
| *Treatment* | |
| Surgery of primary tumour | |
| Modified mastectomy | 180 |
| Breast-conserving lumpectomy | 65 |
| Biopsy only | 4 |
| Surgery of the axilla | |
| Dissection | 239 |
| None | 10 |
| Radiotherapy | |
| Breast or thoracic wall | 143 |
| Axilla | 180 |
| Other lymph node areas | 192 |
| Systemic adjuvant therapy | |
| Chemotherapy (CMF) | 94 |
| Hormonal therapy | 20 |

Owing to a few missing patients characteristics, the numbers of patients do not always add up to 249.

### *Follow-up*

All patients were routinely examined every 3-6 months during the first 5 years and once a year thereafter (median follow-up 49 months; range 23-128 months). Of the 249 patients included in this study, 93 have died, 80 after a previous relapse. In 127 patients recurrence of disease was detected during follow-up, and these patients count as failures in the analysis of disease-free survival.

### *Assay of steroid receptors, UPA and TPK*

Processing and pathological examination of tumours was performed as described previously (Foekens *et al.*, 1989). Tumour tissue was stored in liquid nitrogen. Cytosols were prepared in 10 mM dipotassium hydrogen phosphate buffer [containing 1.5 mM dipotassium chloride EDTA, 3 mM sodium azide, 10 mM monothioglycerol and 10% (v/v) glycerol, pH 7.4]. The values of the cytosolic parameters were not adjusted for histologically assessed percentage of tumour content.

For routine binding assays of cytosolic oestradiol receptors (ER) and progesterone receptors (PR), the dextran-coated charcoal method with multiple-point Scatchard analysis was used according to the procedures as recommended by the European Organization for Research and Treatment of Cancer (EORTC, 1980). The cut-off point used for both was 10 fmol $mg^{-1}$ protein. The same cytosols were used for the assessment of UPA and TPK.

UPA antigen was measured by enzyme-linked immunosorbent assay, as described previously in detail (Foekens *et al.*, 1992), using reagents of a kit which is now commercially available (uPA Elisa kit A894, American Diagnostica, Greenwich, USA).

TPK enzyme activity was determined as previously described (Bolla *et al.*, 1993), using [$^{32}$P]ATP as the phosphate donor and poly(glutamic acid-tyrosine) (4:1) as an artificial substrate. Briefly, an aliquot of the cytosol (20 μg of protein) was incubated in the presence of 5 mM [γ-$^{32}$P]ATP (specific activity 3,000 Ci $mmol^{-1}$), 240 μg $ml^{-1}$ artificial substrate poly-Glu-Tyr (4:1) (Sigma) in 20 mM HEPES buffer, pH 7.5, containing 1 mM EDTA, 1 mM EGTA, 1 mM DTT, 60 μM sodium vanadate, 5 mM sodium fluoride and 8.5 mM manganese chloride. The phosphorylation reaction was run at 20°C for 20 min and stopped upon addition of 1 ml of 10% trichloracetic acid. The mixture was passed through GF/C Whatman filters, which were counted for their radioactivity content, following 2 × 10 ml washes with 10% trichloracetic acid. TPK activity was corrected for endogenous phosphorylation measured in the absence of substrate. TPK levels are expressed in pmol ATP $min^{-1}$ $mg^{-1}$ protein.

### *Statistics*

Linear regression analysis was used to study the correlations between TPK and patients and tumour characteristics. In univariate regression analysis, a highly significant trend ($P = 0.005$) between the log of TPK concentration and the rate of relapse was observed. Subsequently, isotonic regression analysis (Barlow *et al.*, 1972) was applied to check whether a dichotomy or a more or less continuous trend was present. For UPA, a cut-off value of 1.15 ng $mg^{-1}$ protein, as previously determined in a larger breast cancer population was used (Foekens *et al.*, 1992). Disease-free and overall survival probabilities were calculated by the method of Kaplan-Meier. The likelihood ratio test in the univariate Cox regression model was used to test for differences and trend. The Cox proportional hazard model was also applied for multivariate analyses. The results of the Cox regression analysis are summarised by the relative relapse and relative death rates. For the Cox regression analyses, the complete follow-up data were used. The survival curves were limited to 5 years.

### Results

### *Association between TPK and patient and tumour characteristics*

Cytosolic TPK activity varied from 0 to 35.9 pmol ATP $min^{-1}$ $mg^{-1}$ protein (median 11.4; mean ± s.d. 12.1 ± 6.8). No correlation was found with tumour size, number of positive lymph nodes or differentiation grade (Table II). TPK levels were negatively associated with age and menopausal status of the patients. In addition, higher concentrations of TPK were found in ER-negative and PR-negative tumours as compared with receptor-positive tumours. Finally, a positive correlation was found between TPK and UPA.

### *Associations of TPK and other factors with (disease-free) survival*

Table III shows the factors statistically associated with disease-free and overall survival. Age and menopausal status showed no association. Larger tumour size was associated with a reduced disease-free survival. The number of positive lymph nodes was positively associated with both a shorter disease-free and overall survival. ER-PR positivity was associated with a favourable prognosis, whereas high levels of UPA were associated with reduced disease-free and overall survival.

In a test for trend in Cox univariate analysis, logarithmically transformed TPK levels were negatively associated with disease-free survival ($P = 0.005$). Isotonic regression analysis showed a more or less continuous association between the relapse rate and the value of TPK. The median value was therefore arbitrarily chosen as cut-off point to discriminate high- and low-TPK tumours to enable a graphical display with survival curves. Kaplan-Meier curves

**Table II** Relation between TPK and patient and tumour characteristics

| *Characteristics* | n | *Mean* | *s.d.* | *>11.4 (%)*[a] | P-*value* |
|---|---|---|---|---|---|
| Total | 249 | 12.1 | 6.8 | 50.0 | |
| Age (years) | | | | | |
| ≤50 | 80 | 13.7 | 7.8 | 57.5 | |
| 50–65 | 93 | 11.5 | 6.4 | 47.3 | 0.01 |
| >65 | 76 | 11.2 | 5.9 | 44.7 | |
| Post-menopausal | | | | | |
| No | 85 | 13.5 | 7.8 | 56.5 | <0.05 |
| Yes | 164 | 11.4 | 6.2 | 46.3 | |
| Tumour size | | | | | |
| T1 | 61 | 12.1 | 6.9 | 45.9 | |
| T2 | 127 | 12.6 | 6.9 | 52.8 | NS |
| T3 | 33 | 10.3 | 6.9 | 39.4 | |
| T4 | 25 | 11.9 | 6.2 | 56.0 | |
| No. of positive nodes | | | | | |
| 1–3 | 133 | 12.0 | 6.4 | 51.1 | NS |
| >3 | 116 | 12.2 | 7.3 | 48.3 | |
| Grade | | | | | |
| Well[b] | 2 | 6.2 | 5.4 | 0.0 | |
| Moderately | 50 | 12.2 | 5.8 | 50.0 | NS |
| Poorly | 134 | 12.7 | 7.3 | 54.5 | |
| ER status | | | | | |
| – | 61 | 14.4 | 8.4 | 62.3 | <0.01 |
| + | 188 | 11.4 | 6.1 | 45.7 | |
| PR status | | | | | |
| – | 84 | 13.4 | 7.7 | 57.1 | <0.05 |
| + | 156 | 11.6 | 6.2 | 46.1 | |
| UPA status | | | | | |
| – | 162 | 11.4 | 6.8 | 46.3 | <0.05 |
| + | 87 | 13.4 | 6.7 | 56.3 | |

s.d., standard deviation; *P*-value by linear regression analysis. [a]Median value in pmol ATP $min^{-1}$ $mg^{-1}$ protein. [b]Only $n = 2$.

**Table III** Actuarial probabilities of 5 year disease-free and overall survival of patients, as stratified by patient and tumour characteristics

| | *DFS* | | *OS* | |
|---|---|---|---|---|
| | % | P | % | P |
| Age (years) | | | | |
| ≤50 | 54 | | 68 | |
| 50–65 | 40 | NS | 62 | NS |
| >65 | 38 | | 49 | |
| Post-menopausal | | | | |
| No | 55 | NS | 68 | NS |
| Yes | 38 | | 55 | |
| Tumour size | | | | |
| T1 | 59 | | 73 | |
| T2 | 49 | <0.001 | 58 | NS |
| T3 | 24 | | 58 | |
| T4 | 0 | | 37 | |
| No. of positive nodes | | | | |
| 1–3 | 53 | <0.0001 | 70 | <0.0001 |
| >3 | 32 | | 45 | |
| Grade | | | | |
| I[a] | 100 | | 100 | |
| II | 46 | NS | 71 | NS |
| III | 43 | | 56 | |
| ER–PR status | | | | |
| +/+ | 47 | <0.05 | 69 | 0.001 |
| Others[b] | 38 | | 42 | |
| UPA status | | | | |
| – | 51 | <0.0001 | 64 | <0.05 |
| + | 29 | | 50 | |
| TPK status | | | | |
| Low | 50 | 0.01 | 65 | <0.05 |
| High | 37 | | 53 | |

DFS, disease-free survival; OS, overall survival; *P*, *P*-value; NS, not significant. [a]Only $n = 2$. [b]The combined group of the three other phenotypes with one or both receptors negative.

for actuarial disease-free (Figure 1a) and overall survival (Figure 1b) show the increased hazard rates of our patients with TPK-positive primary tumours.

### *Cox multivariate analysis of TPK status*

The independent predictive value of TPK on the rate of relapse and death was assessed with Cox multivariate analysis including various clinical and histological parameters (age, menopausal status, tumour size, number of positive lymph nodes), ER–PR status and UPA (Table IV). Two hundred and thirty-seven patients with complete data were analysed. For disease-free survival, the dominant factors selected in the standard model were tumour size and the number of positive lymph nodes. For overall survival, relevant variables were the number of positive lymph nodes, ER–PR status and tumour size. For both analyses, UPA status (+ *vs* –) and TPK status (high *vs* low) significantly added to the model with relative relapse rates of 2.18 and 1.74 and relative death rates of 1.71 and 1.58, as shown by Kaplan–Meier curves for disease-free (Figure 2a) and overall survival (Figure 2b). Addition of adjuvant chemotherapy as an indicator variable to the multivariate models did not affect the estimates of the regression coefficients of the cytosolic parameters and was therefore not included in the models presented in Table IV.

### Discussion

Several growth factor receptors and oncoproteins, including EGFR (Sainsbury *et al.*, 1987), IGF-1R (Foekens *et al.*, 1989; Peyrat *et al.*, 1990) and HER-2 (Slamon *et al.*, 1987), have been reported in breast tumours. At present, however, there is little agreement on some clinical relationship and their prognostic value (Berns *et al.*, 1992; Klijn *et al.*, 1992). Lack of a standardised assay is probably one of the most important reasons for the variability observed (Koenders *et al.*, 1992; Spyratos *et al.*, 1994). In addition, a single growth factor receptor or oncoprotein probably cannot reliably account for all the cellular signals that occur during tumour growth, invasion and metastasis (Dougall *et al.*, 1993). TPK activity has been associated with the cytoplasmic domain of many growth factor receptors as well as oncogene products (Cantley *et al.*, 1991; Fisher *et al.*, 1991). TPK measurement might thus result in more accurate prediction of patient clinical behaviour than selective measurement of these proteins.

In this study, cytosolic TPK activity was assessed in tumours from 249 node-positive primary breast cancer patients. Although TPK has been associated with membrane-bound protiens (Cantley *et al.*, 1991; Fisher *et al.*, 1991), measurement of TPK activity in cytosol better separates normal breast tissues, benign breast diseases and breast cancers than membrane-bound TPK activity (Hennipman *et al.*, 1989; Lower *et al.*, 1993). Moreover, assay of cytosols appears to be more appropriate for routine use. Measurements were thus performed in cytosols routinely prepared for steroid receptor measurement. We determined TPK enzyme activity using [$^{32}$P]ATP and a polypeptide artificial substrate (Bolla *et al.*, 1993). An enzyme-linked immunosorbent assay (ELISA) has been recently described for TPK measurement (Schraag *et al.*, 1993). At present, little is known about the correlation between the activity data obtained with enzyme activity assay and antigen measurement by ELISA.

The levels of TPK in our study are in agreement with previously published data using the same assay (Bolla *et al.*, 1993). However, we report a strong negative correlation between TPK and ER levels, a relationship not found previously in pilot studies with few patients (Hennipman *et

**Table IV** Cox multivariate analysis of clinical, histological and biological factors

| | Disease-free survival Relative relapse rate (95% confidence limits) | P | Overall survival Relative death rate (95% confidence limits) | P |
|---|---|---|---|---|
| Age and menopausal status[a] | | NS | | NS |
| Age premenopausal[b] | 0.72 (0.46–1.15) | | 0.71 (0.40–1.24) | |
| Age post-menopausal[b] | 0.90 (0.69–1.17) | | 1.24 (0.94–1.65) | |
| Menopausal status[c] | 2.26 (1.15–4.41) | | 1.51 (0.65–3.48) | |
| Tumour size[d] | 1.54 (1.25–1.88) | $<0.0001$ | 1.27 (1.00–1.61) | $<0.05$ |
| No. of positive nodes[e] | 1.85 (1.27–2.69) | 0.001 | 1.93 (1.26–2.97) | $<0.01$ |
| ER-PR status[f] | 0.85 (0.58–1.24) | NS | 0.57 (0.37–0.86) | 0.01 |
| UPA status[g] | 2.18 (1.50–3.16) | $<0.0001$ | 1.71 (1.12–2.63) | 0.01 |
| TPK status[h] | 1.74 (1.18–2.56) | 0.005 | 1.58 (1.02–2.46) | $<0.05$ |

*P*, *P*-value. [a]Age and menopausal status combined. [b]Age in decades tested separately for premenopausal (= pre- + perimenopausal) and post-menopausal patients. [c]Post-menopausal *vs* pre-/perimenopausal. [d]Scored as 1 = T1 to 4 = T4. [e]$>3$ *vs* 1–3. [f]ER and PR positive compared with the combined group of the three other phenotypes with one or both receptors negative. [g]UPA positive as compared with UPA negative. [h]TPK high as compared with TPK low.

**Figure 1** Actuarial disease-free survival **a**, and overall survival **b**, curves stratified by TPK status. The cut-off value for TPK was 11.4 pmol ATP $min^{-1}$ $mg^{-1}$ protein. Numbers represent the number of failures out of the total number of patients in each group.

**Figure 2** Actuarial disease-free survival **a**, and overall survival **b**, curves stratified by both UPA and TPK status. The cut-off value for UPA was 1.15 ng $mg^{-1}$ protein. The cut-off value for TPK was 11.4 pmol ATP $min^{-1}$ $mg^{-1}$ protein. Numbers represent the number of failures out of the total number of patients in each group.

*al.*, 1989; Durocher & Chevalier, 1990; Bolla *et al.*, 1993; Lower *et al.*, 1993). This relationship is similar to that described for EGFR (Foekens *et al.*, 1989; Klijn *et al.*, 1992) and HER-2 (Berns *et al.*, 1992). However, TPK activity has been associated with many growth factor receptors and oncoproteins. Moreover, at least 70% of the TPK activity in breast cancer cytosols originates from the presence of the c-*src* oncogene product (Ottenhoff-Kalff *et al.*, 1992), and the contribution of EGFR to TPK activity seems to be low (Durocher & Chevalier, 1990). In this study, high TPK levels were negatively associated with age and menopausal hormonal status of the patients. Finally, a positive correlation was found between TPK and UPA, which suggests that protease expression may be regulated by growth factors of the TPK family (Laiho & Keski-ga, 1989; Neidbala & Sartorelli, 1989).

High levels of TPK were associated with an increased risk of relapse and death. These data concerning disease-free survival are in agreement with the few previously published pilot studies, which included 18 patients (Hennipman *et al.*, 1989), 86 patients (Lower & Williams, 1992) and 40 node-negative and 40 node-positive patients (Bolla *et al.*, 1993).

TPK retained its prognostic importance in Cox multivariate analysis, including classical clinical and histological factors, steroid receptors and UPA. However, the protease expression, which has been related mainly to invasion and metastasis, appeared as a stronger prognostic factor. ER and PR were stronger predictors of overall survival than of disease-free survival, probably because of the better response to palliative hormonotherapy of ER-positive patients (Nomura *et al.*, 1992). In contrast, disease-free survival and overall survival were equally affected by TPK. The relationship between TPK and sensitivity to adjuvant therapy needs to be clarified. In this study, it could not accurately be assessed because adjuvant therapy was predominantly given to premenopausal patients. Interestingly, however, addition of adjuvant therapy to the multivariate model did not affect the estimates of the cytosolic parameters, including that of TPK.

Assessment of TPK could be helpful for the identification of patients at high risk of recurrence, and thus for the selection of patients for adjuvant therapy. However, the prognostic value of TPK must be confirmed in prospective studies. Measurement of TPK activity might in addition become important in predicting response to TPK inhibitors, of which many are likely to be tested in clinical trials in the near future.

We wish to thank Dr M. Schmitt and Dr F. Jänicke (Munich, Germany) for uPA reagents. This study was supported by the Dutch Cancer Society (Project No. DDHK 92-04).

### References

BARLOW, R.E., BARTHOLOMEW, D.J., BREMNER, J.M. & BRUNK, H.D. (1972). In *Statistical Interference under Order Restrictions*. John Wiley: London.

BERNS, E.M.J.J., KLIJN, J.G.M., VAN PUTTEN, J.G.M., VAN STAVEREN, I.L., PORTENGEN, H. & FOEKENS, J.A. (1992). c-myc amplification is a better prognostic factor than HER2/neu amplification in primary breast cancer. *Cancer Res.*, **52**, 1107–1113.

BOLLA, M., ROSTAING-PUISSANT, B., CHEDIN, M., SOUVIGNET, C., MARRON-CHARRIERE, J., COLONNA, M., BERLAND, E. & CHAMBAZ, E.M. (1993). Protein tyrosine kinase activity as a prognostic parameter in human breast cancer. *Breast Cancer Res. Treat.*, **26**, 283–287.

CANTLEY, L.C., AUGER, K.R., CARPENTER, C., DUCKWORTH, B., GRAZIANI, A., KAPELLER, R. & SOLTOFF, S. (1991). Oncogenes and signal transduction. *Cell*, **64**, 281–302.

DOUGALL, W.C., QIAN, X. & GREENE, M.I. (1993). Interaction of the Neu/p185 and EGF receptor tyrosine kinases: implications for cellular transformation and tumor therapy. *J. Cell. Biochem.*, **53**, 61–73.

DUROCHER, Y. & CHEVALIER, S. (1990). Protein tyrosine kinases in human breast cancer: kinetic properties and evidence for the presence of two forms of native enzyme. *Breast Cancer Res. Treat.*, **17**, 99–107.

DVIR, A., MILNER, Y., CHOMSKY, O., GILON, C., GAZIT, A. & LEVITZKI, A. (1991). The inhibition of EGF-dependent proliferation of keratinocytes by tyrphostin tyrosine kinase blockers. *J. Cell. Biol.*, **113**, 857–865.

ENNIS, B., LIPPMAN, M.E. & DICKSON, R.B. (1991). The EGF receptor system as a target for antitumor therapy. *Cancer Invest.*, **9**, 553–562.

EORTC BREAST CANCER COOPERATIVE GROUP (1980). Revision of the standards for the assessment of hormone receptors in human breast cancer. *Eur. J. Cancer*, **16**, 1513–1515.

FISHER, E.H., CHARBONNEAU, H. & TONKS, N.K. (1991). Signal transduction by receptors with tyrosine kinase activity. *Science*, **253**, 401–406.

FOEKENS, J.A., PORTENGEN, H., VAN PUTTEN, W.L.J., TRAPMAN, A.M.A.C., RUEBI, J.C., ALEXIEVA-FIGUSCH, J. & KLIJN, J.G.M. (1989). Prognostic value of receptors for insulin-like growth factor 1, somatostatin, and epidermal growth factor in human breast cancer. *Cancer Res.*, **49**, 7002–7009.

FOEKENS, J.A., SCHMITT, M., VAN PUTTEN, W.L.J., PETERS, H.A., BONTENBAL, M., JÄNICKE, F. & KLIJN, J.G.M. (1992). Prognostic value of urokinase-type plasminogen activator in 671 primary breast cancer patients. *Cancer Res.*, **52**, 6101–6105.

HARRIS, J.R., LIPPMAN, M.E., VERONESI, U. & WILLETT, W. (1992). Medical progress. Breast cancer (third of three parts). *N. Engl. J. Med.*, **327**, 390–398.

HENNIPMAN, A., VAN OIRSCHOT, B.A., SMITS, J., RIJKSEN, G. & STAAL, G.E.J. (1989). Tyrosine kinase activity in breast cancer, benign breast disease and normal breast tissue. *Cancer Res.*, **49**, 516–521.

KLIJN, J.G.M., BERNS, P.M.J.J., SCHMITZ, P.I.M. & FOEKENS, J.A. (1992). The clinical significance of epidermal growth factor receptor (EGF-R) in human breast cancer: a review on 5232 patients. *Endocrine Rev.*, **13**, 3–17.

KOENDERS, P.G., FAVERLY, D., BEEX, L.V.A.M., BRUGGINK, E.D.M., KIENBUIS, C.B.M. & BENRAAD, T.J. (1992). Epidermal growth factor receptors in human breast cancer: a plea for standardisation of assay methodology. *Eur. J. Cancer*, **28**, 693–697.

LAIHO, M. & KESKI-OJA, J. (1989). Growth factors in the regulation of pericellular proteolysis: a review. *Cancer Res.*, **49**, 2533–2553.

LOWER, E.E. & WILLIAMS, L. (1992). Phosphotyrosine expression in breast cancer specimens is associated with worse prognosis. *Proc. Am. Assoc. Cancer Res.*, **33**, 373.

LOWER, E.E., FRANCO, R.S., MILLER, M.A. & MARTELO, O.J. (1993). Enzymatic and immunohistochemical evaluation of tyrosine phosphorylation in breast cancer specimens. *Breast Cancer Res. Treat.*, **26**, 217–224.

NIEDBALA, M.J. & SARTORELLI, A.C. (1989). Regulation by epidermal growth factor of human squamous cell carcinoma plasminogen activator-mediated proteolysis of extracellular matrix. *Cancer Res.*, **49**, 3302–3309.

NOMURA, Y., MIURA, S., KOYAMA, H., ENOMOTO, K., KASUMI, F., YAMAMOTO, H., KIMURA, M., TOMINAGA, T., IINO, H., MORIMOTO, T. & TASHIRO, H. (1992). Relative effect of steroid receptors on the prognosis of patients with operable breast cancer. *Cancer*, **69**, 153–164.

OTTENHOFF-KALFF, A.E., RIJKSEN, G., VAN BEURDEN, A.A.C.M., HENNIPMAN, A., MICHELS, A.A. & STAAL, G.E.J. (1992). Characterization of protein tyrosine kinases from human breast cancer: involvement of the c-src oncogene product. *Cancer Res.*, **52**, 4773–4778.

PEYRAT, J.P., BONNETERE, J., VENNIN, P.H., JAMMES, H., BEUSCART, R., DJIANE, J., LEFEVRE, J. & DEMAILLE, A. (1990). Insulin-like growth factor 1 receptors (IGF1-R) and IGF1 in human breast tumors. *J. Steroid Biochem. Mol. Biol.*, **37**, 823–827.

SAINSBURY, J.R.C., NEEDHAM, G.K., FARNDON, J.R., MALCOLM, A.J. & HARRIS, A.L. (1987). Epidermal-growth factor receptor status as predictor of early recurrence and death from breast cancer. *Lancet*, **i**, 1398–1402.

SCHRAAG, B., STAAL, G.E.J., ADRIAANSEN-SLOT, S.S., SALDEN, M. & RIJKSEN, G. (1993). Standardization of an enzyme-linked immunosorbent assay for the determination of protein tyrosine kinase activity. *Anal. Biochem.*, **211**, 233–239.

SLAMON, D.J., CLARK, G.M., WONG, S.G., LEVIN, W.J., ULLRICH, A. & MCGUIRE, W.L. (1987). Human breast cancer: correlation of relapse and survival with amplification of the HER2/neu oncogene. *Science*, **235**, 177–182.

SPYRATOS, F., MARTIN, P.M., HACENE, K., ANDRIEU, C., ROMAIN, S., FLOIRAS, J.L. & MAGDELENAT, H. (1994). Prognostic value of a solubilized fraction of EGF receptors in primary breast cancer using an immunoenzymatic assay – a retrospective study. *Breast Cancer Res. Treat.* (in press).

ULLRICH, A. & SCHLESSINGER, J. (1990). Signal transduction by receptors with tyrosine kinase activity. *Cell*, **61**, 203–212.

WILKS, A.F. (1993). Protein tyrosine kinase growth factor receptors and their ligands in development, differentiation, and cancer. *Adv. Cancer Res.*, **60**, 43–73.